# Augmented drug combination dataset to improve the performance of machine learning models predicting synergistic anticancer effects

**DOI:** 10.21203/rs.3.rs-3481858/v1

**Published:** 2023-10-28

**Authors:** Mengmeng Liu, Gopal Srivastava, J. Ramanujam, Michal Brylinski

**Affiliations:** Louisiana State University; Louisiana State University; Louisiana State University; Louisiana State University

**Keywords:** data augmentation, anticancer drug synergy, machine learning, combination pharmacotherapy, augmented AZ-DREAM Challenges

## Abstract

Combination therapy has gained popularity in cancer treatment as it enhances the treatment efficacy and overcomes drug resistance. Although machine learning (ML) techniques have become an indispensable tool for discovering new drug combinations, the data on drug combination therapy currently available may be insufficient to build high-precision models. We developed a data augmentation protocol to unbiasedly scale up the existing anti-cancer drug synergy dataset. Using a new drug similarity metric, we augmented the synergy data by substituting a compound in a drug combination instance with another molecule that exhibits highly similar pharmacological effects. Using this protocol, we were able to upscale the AZ-DREAM Challenges dataset from 8,798 to 6,016,697 drug combinations. Comprehensive performance evaluations show that Random Forest and Gradient Boosting Trees models trained on the augmented data achieve higher accuracy than those trained solely on the original dataset. Our data augmentation protocol provides a systematic and unbiased approach to generating more diverse and larger-scale drug combination datasets, enabling the development of more precise and effective ML models. The protocol presented in this study could serve as a foundation for future research aimed at discovering novel and effective drug combinations for cancer treatment.

## Background

Developing effective anticancer therapies is an important yet challenging task. Most currently available treatments employ a monotherapy, i.e., using a single drug to treat a particular disease [1, 2]. Although widely used, monotherapies are known to suffer from certain problems, such as the acquired drug resistance and prominent side effects [1, 3]. In contrast, combination therapies utilizing multiple pharmaceuticals to simultaneously target several biological processes generally have greater chances of overcoming these issues [4]. Not surprisingly, combination therapies against complex diseases, such as cancer, are attracting a significant attention. Nonetheless, exploring all possible drug combinations within a vast pharmacological space is a major obstacle to find those drug combinations exhibiting synergistic effects. Accurate computational methods to select the most promising therapeutic candidates for experimental testing can greatly facilitate the discovery of effective drug combinations.

Approaches utilizing machine learning (ML) are well suited to predict drug synergistic effects. Supervised learning techniques require large-scale experimental data to train models predicting effective drug combinations. These datasets differ with respect to the number of drugs and cell lines. For instance, A Large Matrix of Antineoplastic Agent Combinations from the National Cancer Institute (NCI-ALMANAC) contains 5,232 drug pairs tested against 60 cancer cell lines [5]. Another resource provides drug responses measured for a panel of 39 cancer cell lines and 22 experimental drugs in all possible pairwise combinations and in combination with 16 approved drugs, totaling 583 compound pairs [6]. Other datasets are focused on a specific cell line, for example, 1,833 bioactive drugs at 5 μm were tested in combination with temozolomide at 400 μm against a human glioblastoma cell line T98G^N^ [7]. Furthermore, 1,327 drug combinations from the CeMM library of unique drugs (CLOUD) dataset containing 308 prodrugs and active drugs [8] were found effective against a human chronic myeloid leukemia cell line KBM-7 [9].

Meta-datasets collect and standardize the results of individual drug combination screening studies in order to enable a more efficient utilization of these data resources. For instance, DrugComb is an open-access data portal to 739,964 combinations of 8,397 drugs tested on 2,320 cell lines from 33 tissues [10, 11]. It quantifies the degree of drug-drug interactions over the full dose-response matrix with several synergy scores, Bliss independence (BLISS), Highest single agent (HSA), Loewe additivity (LOEWE), and Zero interaction potency (ZIP) [12–14]. SYNERGxDB is a comprehensive dataset compiled from nine individual datasets containing 22,507 pairwise combinations of 1,977 drugs tested on 151 cell lines from 15 tissues [15]. Similar to DrugComb, SYNERGxDB also provides standardized synergy scores, BLISS and ZIP. Finally, Dialog for Reverse Engineering Assessments and Methods (DREAM) Challenges partnered with AstraZeneca and the Sanger Institute to compile a dataset of 20,483 synergy scores for 910 drug combinations involving 118 anticancer drugs tested against 85 cancer cell lines [16]. This dataset also provides a quality assessment score for each combination, ranging from −3 to 1, where 1 indicates a synergy between drugs in the combination. Along with the synergy data for drug combinations, the AZ-DREAM Challenges data comprise various molecular data, such as mutations, copy number variation, gene expression, and the tissue of origin. These datasets offer unparalleled opportunities to develop highly accurate ML models to predict drug synergistic effects.

Since the performance of supervised ML strongly depends on the quality, quantity, and the contextual subject of training data, the data scarcity problem is one of the most common challenges to develop robust ML models. To overcome this difficulty, data augmentation techniques are widely employed to expand the volume of available data. For instance, classical augmentation methods, such as image flipping, image rotation, noise injection, kernel filters, random erasing, and image mixing, are frequently used in the medical image analysis domain [17–22]. Data augmentation techniques gaining attention in the medical time series analysis domain [23] include the time domain augmentation [24], the time-frequency domain augmentation [25], decomposition-based methods [26, 27], statistical generative models [28, 29], and learning-based methods [30–33]. In addition, more advanced deep learning-based augmentation techniques, including the feature space augmentation [34, 35], generative adversarial networks (GAN)-based augmentation [36–40], the neural style transfer [41, 42], and meta-learning schemes [43–45], have been proposed.

Although image and sequential data augmentation methods are well established, these approaches are, in principle, unsuitable to generate the heterogeneous data of cellular and molecular features for drug synergy prediction with supervised ML. On that account, a variety of domain-specific techniques have been developed. For instance, the fact that multiple simplified molecular-input line-entry system (SMILES) strings represent the same molecule was used to augment a molecular dataset of chemical species [46] using the SMILES enumeration [47]. Further, the size of a training dataset to predict anticancer drug synergism based on NCI-ALMANAC was doubled by generating duplicates with the reverse order of drugs [48]. Another method applies the data up-sampling to increase the number of minor class instances for phenotype-based virtual screening of anticancer drug combinations [49]. An example of a deep learning-based data augmentation technique is the uniform graph convolutional network (UGCN) [50]. It employs a drug representation based on atomic interactions within organic compounds rather than hand-crafted features, such as molecular fingerprints, and string-based features, such as SMILES. UGCN can be used to augment chemical data by randomly sampling multiple complementary graphs for a single drug.

Despite the encouraging results reported for the abovementioned data augmentation techniques for drug synergy prediction, many of existing methods either are too general (up-sampling) or consider only drug structural information (SMILES enumeration and UGCN). To address these issues, we devised a new augmentation approach combining the drug chemical similarity with the system-level information on drug-target interactions. This approach employs a novel similarity metric, the drug action/chemical similarity (DACS) score, taking into account not only the chemical characteristics of drugs, but also their molecular targets. Applying the DACS score to augment the AZ-DREAM Challenges data with new compounds from PubChem [51] significantly increased the size and diversity of the training dataset for drug synergy prediction. To the best of our knowledge, this methodology represents the first systematic and effective protocol to augment a synergy dataset simultaneously utilizing the information on drug chemical structures and their protein targets. As a proof of concept, the augmented dataset was used to train several ML models demonstrating a higher accuracy of drug synergy prediction compared to those models trained on the original AZ-DREAM Challenges data.

## Results

### Similarity measure for cellular responses to drug treatment

**Figure 1:**
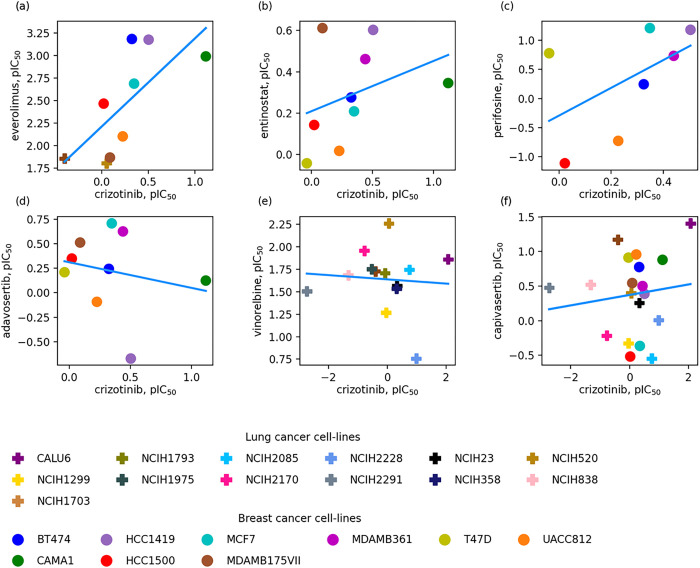
Similarity of pharmacological effects of two drugs quantified by the Kendall τ correlation coefficient. pIC_50_ values for the monotherapy treatments of multiple cancer cell lines with crizotinib are plotted against those for (**A**) everolimus, (**B**) entinostat, (**C**) perifosine, (**D**) adavosertib, (**E**) vinorelbine, and (**F)** capivasertib. **A, B,** and **C** are examples of the positive correlation, whereas **D, E,** and **F** represent the negative correlation. Individual breast cancer cell lines are shown as solid circles and lung cancer cell lines as solid plus signs.

During the data augmentation, new drug combinations are generated by replacing drugs with those molecules triggering similar pharmacological responses. The similarity of pharmacological effects of two drugs is quantified by the Kendall τ correlation coefficient between pIC_50_ values for the monotherapy treatments of multiple cancer cell lines. A positive value of Kendall τ indicates that two drugs have similar pharmacological effects in terms of the inhibition of the cancer growth, whereas a negative correlation and the lack of correlation point to different cellular responses to drug treatment. This concept is illustrated in Fig. 1 for crizotinib, a tyrosine kinase inhibitor used for the treatment of non-small cell lung carcinoma (NSCLC) [52], paired with six other anti-cancer drugs. Figures 1A, 1B, and 1C are examples of a positive correlation between crizotinib and everolimus (Kendall τ of 0.50), entinostat (Kendall τ of 0.44), and perifosine (Kendall τ of 0.42), respectively. Everolimus, a derivative of sirolimus with cell proliferation and immunosuppressive properties, is used in combination with other anticancer agents for the treatment of kidney and breast cancer, and neuroendocrine tumors of gastrointestinal and lung origins [53]. Entinostat, a benzamide derivative with the antineoplastic activity, and perifosine, an allosteric AKT inhibitor with the antiglycolytic activity, are used for the treatment of NSCLC [54, 55]. According to the analysis of pIC_50_ values against multiple cancer cell lines, these three drugs have similar profiles to that of crizotinib, i.e., they inhibit the growth of the same cancer cell lines and are ineffective against the same group of cell lines as well.

In contrast, cellular responses of crizotinib are uncorrelated with that of adavosertib (Fig. 1D, Kendall τ of −0.06), vinorelbine (Fig. 1E, Kendall τ of −0.03), and capivasertib (Fig. 1F, Kendall τ of −0.01). Adavosertib is a tyrosine kinase WEE1 inhibitor used to improve the outcome in triple-negative breast cancer [56], vinorelbine is an agent to treat NSCLC and breast cancer [57], and capivasertib is AKT inhibitor used in the treatment of breast cancer [58]. Since these drugs have uncorrelated pharmacological effects, they cannot be used to replace crizotinib during the data augmentation process. The analysis of cellular responses with the Kendall τ is versatile and can be applied when two drugs have been tested on at least two common cell lines, otherwise the value of the Kendall τ is set to 0. The similarities of pharmacological effects between crizotinib and everolimus, entinostat, perifosine, adavosertib, vinorelbine, and capivasertib were calculated based on 7 + 2, 9 + 0, 7 + 0, 9 + 0, 0 + 13, and 9 + 10 common (breast + lung) cell lines, respectively.

### Relation between drug similarity and pharmacological effects

**Figure 2:**
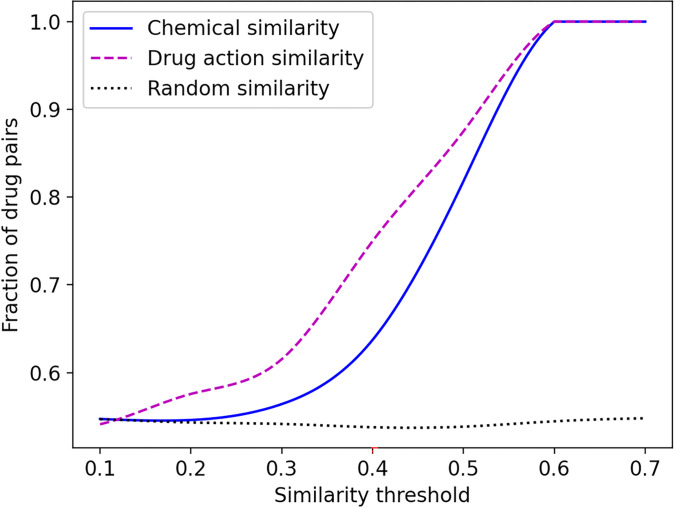
Fraction of drug pairs with positively correlated pharmacological effects as a function of their similarities. The chemical similarity (solid blue line) is measured with the Tanimoto coefficient between drug FP2 fingerprints. The drug action similarity (dashed purple line) is quantified with the Matthews correlation coefficient between target proteins in the IHP-PING protein-protein interaction network. Random similarity (dotted black line) is obtained by assigning a random number between 0 and 1.

Next, we investigate how similar two drugs need to be in order to trigger similar pharmacological effects. This analysis is performed for 4,753 (98C2) possible pairs of 98 drugs in the AZ-DREAM Challenges dataset. Pharmacological responses are quantified with the Kendall τ correlation coefficient, whereas the drug similarity is measured with two metrics. The first score is the drug chemical similarity calculated as the Tanimoto coefficient (TC) between FP2 fingerprints [59]. Figure 2 (solid blue line) shows that, as expected, the fraction of drug pairs with the positive Kendall τ increases with the increasing chemical similarity and reaches a value of 1.0 for the TC threshold of 0.6. The second metric is the drug action similarity computed as the Matthews correlation coefficient (MCC) [60] between target proteins in the protein-protein interaction (PPI) network from the IHP-PING dataset [61]. Similar to the TC, the fraction of drug pairs with the positive Kendall τ also increases with the increasing MCC reaching 1.0 for the MCC threshold of 0.6 (Fig. 2, dashed purple line). For comparison, increasing the threshold for a random similarity does not increase the fraction of drug pairs with the positive Kendall τ (Fig. 2, dotted black line).

### Drug action/chemical similarity score

**Figure 3:**
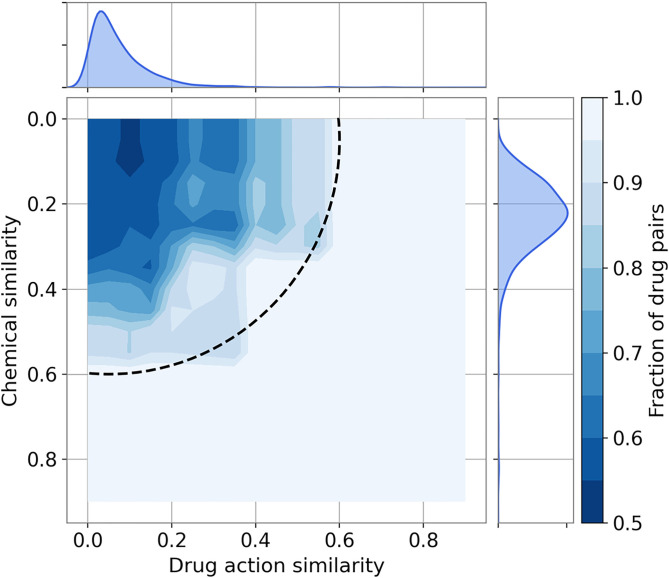
Heatmap of the fraction of drug pairs with positively correlated pharmacological effects. The fraction of drug pairs with the positive Kendall τ is displayed according to the color scale on the right. One-dimensional histograms show the distributions of the chemical similarity (a subplot on the right) and the drug action similarity (a subplot on the top). The dashed quarter circle represents a DACS threshold of 0.6.

Analyses presented above demonstrate that both chemical and drug action similarities can be used for data augmentation. However, their combination could potentially cover a larger chemical space than individual similarities while ensuring that the pharmacological profiles of drugs selected for augmentation are highly similar to those of their parent molecules. Therefore, we combined TC and MCC into a new metric, the drug action/chemical similarity (DACS) score. Figure 3 shows the relation between the DACS score and the fraction of drug pairs with the positive Kendall τ as the spatial heatmap in two dimensions corresponding to the individual similarities. The dark blue section in the upper left corner of the heatmap corresponds to the area of a low positive correlation, whereas the light blue section shows the combination of individual similarities resulting in a high positive correlation. The DACS score can be represented as a quarter circle in Fig. 3 (dashed black line). For example, above a DACS threshold of 0.6, as many as 85.7% drug pairs have a positive Kendall τ correlation.

### Dataset augmentation with DACS

**Figure 4:**
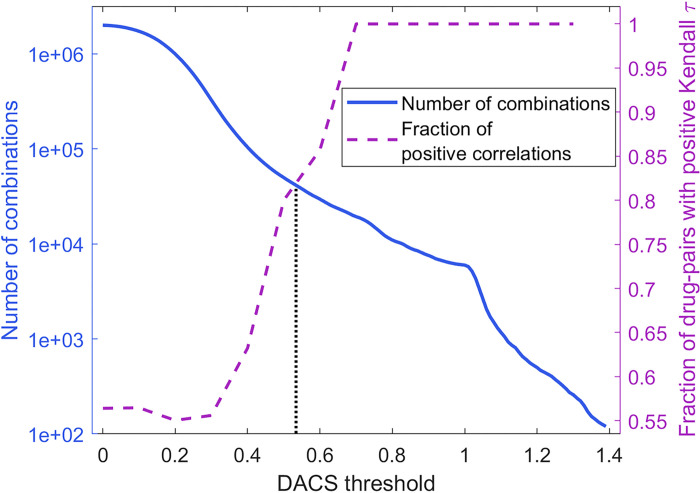
Selection of the optimal DACS threshold for data augmentation. The solid blue curve represents the number of potential substitutes for the original 98 drugs that can be found in the STITCH database as the DACS threshold is increased. The dashed purple line represents the change in the fraction of drug pairs with the positive Kendall τ as the DACS threshold is increased. The vertical dotted line marks the DACS threshold optimizing these two quantities.

The DACS metric is used as a guide to find the optimal number of new instances to be generated for the synergy dataset. Each instance in the AZ-DREAM Challenges dataset consists of a pair of drugs targeting a cell line with a particular synergy score. During the augmentation procedure, one drug in a pair is replaced by a new drug selected from the STITCH database [62]. First, DACS scores between the original drug to be replaced and the candidate substitute compounds were calculated for the synergy dataset. Then, we conducted an analysis of the fraction of new drugs having similar pharmacological profiles to their parent molecules and the number of new instances that can be obtained from the STITCH database at different DACS similarity thresholds. Figure 4 shows that these two quantities are inversely related, i.e., increasing the DACS similarity threshold results in a higher chance of substitute compounds to trigger similar pharmacological responses (dashed purple line), however, at the same time, fewer molecules can be used to augment the dataset (solid blue line).

**Figure 5:**
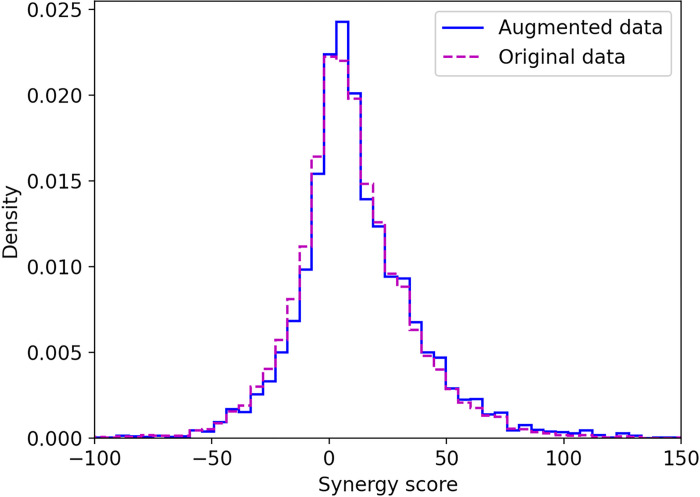
Distribution of synergy score across drug synergy datasets. The step histogram in purple dashed line shows the distribution of synergy scores in the original AZ-DREAM Challenges data, whereas the step histogram in blue solid line shows the distribution of synergy scores in the augmented dataset.

The intersection point marked by a dotted black line in Fig. 4 represents the optimal DACS cutoff of 0.53, at which the majority of substitute drugs (82%) have similar pharmacological profiles to their parent molecules and as many as 42,225 new drugs can be obtained from the STITCH database to augment the synergy dataset. Applying this threshold to replace one molecule in a drug pair in the AZ-DREAM Challenges dataset of 8,798 instances produces an augmented dataset of 6,016,697 drug pairs annotated with synergy scores against various cancer cell lines. Ideally, the distribution of synergy values across the augmented dataset should be the same as for the AZ-DREAM Challenges dataset. Figure 5 shows that these two distributions indeed are similar; the average synergy score ±standard deviation is 9.9 ±26.1 for the AZ-DREAM Challenges dataset and 12.1 ±28.5 for the augmented dataset. This analysis demonstrates that the augmented dataset does not contain artifacts that could potentially bias the training of machine learning models toward a particular effect (either synergism or antagonism).

### Drug synergy prediction with machine learning

Finally, we investigate whether training machine learning against the augmented data achieves a better classification performance than training against the original AZ-DREAM Challenges dataset. Two state-of-the-art machine learning models are implemented employing Random Forest (RF) [63] and Gradient Boosting Trees (GBT) [64] classifiers. Following the original publication [16], drug pairs having synergy scores higher than 20 are labelled synergistic and those having synergy scores lower than −20 are labelled antagonistic. First, we performed a 5-fold cross-validation by randomly splitting the dataset into 5 subsets. Note that the augmented data are only used to train machine learning models, which are then validated against AZ-DREAM Challenges instances. Table 1 shows the classification performance evaluated with several metrics. Encouragingly, the performance of both classifiers is improved when models are trained against the augmented data and the random-split validation is employed. For instance, the MCC increased from 0.39 to 0.40 for RF and from 0.50 to 0.52 for GBT classifiers.

Although a random-split cross-validation is often used to assess the performance of drug synergy predictors [16], it leads to a significant overlap between training and validation subsets because those instances involving similar cell lines are present in both sets. Consequently, the trained model is going to have only a weak ability to generalize to unseen data, even though the validation accuracy may seem high. In order to mitigate this issue and more reliably evaluate the performance of machine learning trained on drug synergy data, we conducted a tissue-based cross-validation in which each fold comprises a particular tissue (or a group of tissues). This protocol has been shown to eliminate the overlap between training and validation subsets allowing for an unbiased assessment of the capabilities of machine learning to extract the information from input data [65].

**Figure 6:**
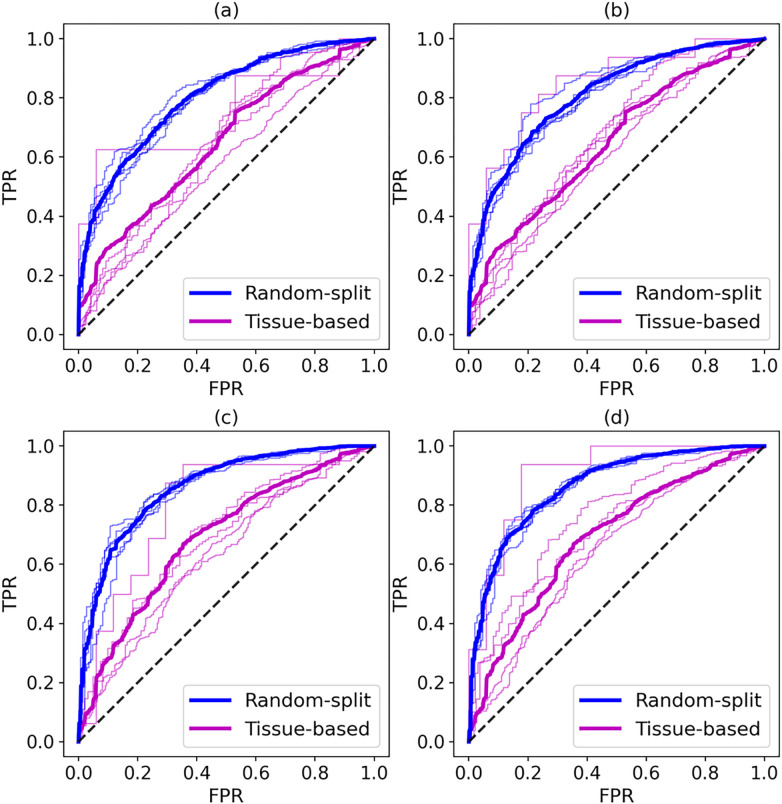
Performance of machine learning in the prediction of drug synergistic effects. Receiver operating characteristics plots for the Random Forest classifier against (**A**) the original AZ-DREAM Challenges data and (**B**) the augmented dataset, and for the Gradient Boosting Trees classifier against (**C**) the original AZ-DREAM Challenges data and (**D**) the augmented dataset. Blue lines were calculated for the random-split protocol, while purple lines were obtained for the tissue-based cross-validation. Thick lines show the mean performance averaged over individual folds represented by thin lines.

Table 1 and receiver operating characteristic plots presented in Fig. 6 show that applying the more rigorous tissue-based validation protocol decreases the performance of machine learning predicting drug synergistic effects. However, this evaluation is more reliable because it better mimics a real scenario in which machine learning is applied to predict drug synergistic effects for unseen data, i.e., drug combinations against cell lines originating from tissues that have not been used to train the classifier. With this cross-validation protocol, machine learning trained on the augmented data yields even higher improvements in terms of the classification accuracy compared to models trained on the original AZ-DREAM Challenges dataset. For example, the MCC increased from 0.173 to 0.226 for RF and from 0.176 to 0.260 for GBT classifiers. Table 2 shows the area under the receiver operating characteristic plot (AUC) scores for each tissue fold and models trained on both the original and the augmented datasets. The comparison of AUC scores reveals that incorporating the augmented data into the training process systematically improves the classification performance regardless of the tissue type. In general, these findings indicate that incorporating augmented data can provide enhanced information for training machine learning models in a more effective manner.

## Discussion

In this study, we devised a data augmentation protocol to solve the data scarcity problem in predicting synergistic effects of anti-cancer drug combinations with machine learning models. The augmentation protocol expands the synergy dataset by replacing a compound in a drug combination instance with another molecule having highly similar pharmacological effects. This is achieved through the use of the DACS similarity metric between two drugs, which incorporates both chemical structure and drug action similarities. Compared to existing techniques used in synergy data augmentation, such as the upsampling [49], the SMILES enumeration [47], and the reverse order of drugs [48], which essentially duplicate the existing data points, our approach expands the dataset by including new, unbiased instances. As a results, this augmentation methodology not only enriches the available data points, but also enhances the diversity of the data, which is highly beneficial to improve the generalizability of machine learning models. Additionally, in contrast to other augmentation approaches involving a learning process [50], our method generates data points in a shorter amount of time.

While random-split cross-validation is frequently utilized for data partitioning, it may lead to tissue-level overlap and elevate the possibility of model overfitting, particularly when dealing with data containing multiple cell lines from the same tissue. The reason for this is that those instances involving similar cell lines tend to have comparable feature representations, such as gene expression profiles and the gene-disease association. The overlap is likely going to occur when these instances are present in both the training and validation sets [66]. In such cases, the trained model may exhibit a strong performance due to the presence of overlapping data, but it will not perform well on novel, unseen data. Consequently, the model may be overestimated in terms of its true performance and fail to generalize to other datasets. On the other hand, a tissue-based cross-validation can effectively eliminate the data overlap issue. By excluding all instances originating from a validation tissue from the training set for each fold, the generalizability of a machine learning model can be properly evaluated.

Tree-based models (RF and GBT) employed in this study are robust, interpretable, and widely adopted by AZ-DREAM Challenges participants [16]. These models have the ability to deal with complex non-linear input-output relationships and can handle sizable datasets to a certain degree. However, tree-based models are not designed to exploit intricate relationships between features, especially when these features are heterogeneous in nature, including protein-protein interactions, gene expression levels, and drug-protein associations. In such cases, these models may struggle to find the optimal decision boundaries, generally leading to an unsatisfactory performance. Neural networks, on the other hand, are better equipped to handle diverse data types and can learn complex relationships between features with hidden layers and non-linear activation functions. This ability to integrate multiple heterogeneous data into a single model can often result in an improved performance compared to tree-based models. Our future research will concentrate on exploring this aspect.

The augmentation protocol devised in this study is not limited to anti-cancer drug data can be used to expand other synergy datasets as well; it has the potential to become a universal tactic for enhancing datasets in drug discovery and related fields. This could result in a greater amount of data being accessible and ultimately lead to better research results. Furthermore, the developed new drug similarity measure, the DACS score, improves the way drug similarity is assessed. By integrating both structural and target similarities, DACS provides a more exhaustive and inclusive perspective on drug similarity compared to traditional methods that only examine a single aspect, such as the chemical similarity. By offering a more holistic approach to analyzing and evaluating the similarities between drugs, DACS can help improve the accuracy and efficiency of the drug discovery process.

Deep learning, with its ability to dissect complex data and reveal underlying patterns and relationships, has become a pivotal tool in the field of pharmacology and drug development [67, 68]. The varied and comprehensive synergy dataset created in this study has the potential to significantly aid deep learning models by offering a diverse range of data for training purposes. The utilization of sufficient data enables deep learning algorithms to recognize intricate relationships and connections among cellular, molecular, and biological system-level features, thereby elevating the precision and efficacy of synergistic effect predictions. Moreover, an extensive and varied dataset reduces the risk of overfitting, a common issue where models become too reliant on limited training data and struggle to generalize to new data. Thus, the utilization of a comprehensive synergy dataset can lead to more robust and dependable deep learning models and ultimately, more advanced outcomes in drug discovery and related fields.

In addition to being used in deep learning-based drug discovery, the proposed anti-cancer drug synergy dataset has the potential to facilitate other applications, such as drug repositioning, drug target identification, toxicity analysis, the modeling of drug interactions, systems pharmacology, and precision medicine. By providing valuable insights into the interactions between drugs, targets, and biological systems, the synergy data can contribute to the development of more effective and safer pharmaceutics. Overall, the wide-ranging possibilities arising from this study may have significant implications for the drug discovery and development field. Ultimately, this could result in the creation of novel therapeutic approaches for a range of diseases.

## Methods

### Similarity of drug pharmacological effects

The Kendall τ rank correlation coefficient is employed to measure the ordinal association between the pharmacological effects of two drugs against a set of cell lines. First, common cell lines targeted by both drugs are identified and two lists ranked by pIC_50_ values for monotherapy treatments are calculated. Next, the value of the Kendall τ accounting for ties $\left(\tau_{b}\right)$ [69, 70] is computed:

Eq. 1 $$\tau_{b}=\frac{n_{c}-n_{d}}{\sqrt{\left(n_{c}+n_{d}+n_{1}\right)\left(n_{c}+n_{d}+n_{2}\right)}}$$

where $n_{c}$ is the number of concordant cell line pairs (having the same order in both drug lists), $n_{d}$ is the number of discordant cell line pairs (having different order in both drug lists), $n_{1}$ is the number of pairs tied only in the first list, and $n_{2}$ is the number of pairs tied only in the second list. $\tau_{b}$ of +1 indicates a perfectly positive association, i.e., the two drugs having the same pharmacological effects in terms of the inhibition of the cancer growth across multiple common cell lines. A value of −1 indicates a perfectly negative association, i.e., the opposite pharmacological effects, and a value of 0 indicates the lack of any association. The Kendall τ coefficient is calculated when pIC_50_ values are available for monotherapy treatments of at least two common cell lines, otherwise it is set to 0.

### Similarity of drug molecular mechanism of action

Similarity of the mechanism of action of two drugs is quantified with the MCC [60] computed for 19,968 proteins in the IHP-PING dataset [61] according to chemical-protein associations obtained from the STITCH database [62]:

Eq. 2 $$MCC=\frac{\left(T\times N)-(A\times B\right)}{\sqrt{\left(T+A)(T+B)(N+A)(N+B\right)}}$$

where T is the number of proteins targeted by both drugs, N is the number of proteins not targeted by any drug, A is the number of proteins only targeted by the first drug, and B is the number of proteins only targeted by the second drug. MCC ranges from −1 to +1 with high positive values indicating a significant overlap between the molecular targets of two drugs, thus a similar mechanism of action. The MCC for a pair of drugs having different mechanisms of action is going to be around 0.

### Drug action/chemical similarity score

The DACS measure provides a convenient and informative way to combine the drug structure similarity with the similarity of the molecular mechanisms of action. It is calculated as:

Eq. 3 $$DACS=\sqrt{TC^{2}+MCC^{2}}$$

where TC is the Tanimoto coefficient between drug FP2 fingerprints [59] and MCC is the similarity of drug mechanism of action defined in Eq. 1. When one of the component metrics, either TC or MCC, is sufficiently high, then the other metric does not need to be as high for the DACS score to be over a predefined threshold. In rare cases of negative MCC values, the MCC component of the DACS score is set to 0.

### Classification datasets

Removing instances having ambiguous synergy scores between −20 and 20 resulted in a classification dataset of 3,210 drug combinations comprising 2,461 synergistic (a synergy score ≥ 20) and 749 antagonistic (a synergy score ≤ −20) cases. The corresponding augmented dataset contains 1,850,037 synergistic and 465,288 antagonistic combinations totaling 2,315,325 labeled instances.

### Feature vectors

Input data for machine learning consist of drug and cell features. The former are computed with Mol2vec [71] by encoding a drug chemical structure to a 300-dimensional vector. The latter features are calculated by embedding 17,419 gene expression values for a cell line obtained from the AZ-DREAM Challenges dataset with an adversarial deconfounding autoencoder [72]. Similar to drug embeddings, the gene expression profile is encoded to a 300-dimensional vector. The final, 900-dimensional feature vector is generated by concatenating two drug feature vectors and a cell feature vector.

### Cross-validation protocols

Two cross-validation procedures are employed utilizing a random and a tissue-based data split. In the random-split cross-validation, the classification dataset is randomly partitioned into five equal-size folds. In the tissue-based cross-validation, the dataset is assigned to five groups according to the tissue type of cell lines, the breast tissue, the digestive system, the excretory system, the respiratory system, and other tissues. Note that tissue types in the augmented dataset are the same as in the original dataset because the augmentation process does not affect cell lines. A 5-fold cross-validation is conducted the usual way, i.e., in each round, the machine learning model is trained on the augmented data for 4 subsets and then validated against the original AZ-DREAM Challenges instances in the remaining subset. This protocol ensures that the augmented data is used only to train classifiers and the validation is performed on the original data and labels. Since the original dataset is imbalanced, comprising 76.7% synergistic and 23.3% antagonistic instances, a stratified split is used to preserve the percentage of samples for each class in each fold. When augmenting the training set, the ratio is preserved by proportionally adding instances of each class. In the tissue-based split, although the proportions of synergistic and antagonistic instances are different in each tissue, the training set is augmented in a way to preserve the ratio of synergistic/antagonistic instances in individual folds.

### Machine learning

Two machine learning models are used to evaluate the performance of supervised learning algorithms on the original and the augmented datasets of drug combinations, Random Forest and Gradient Boosting Trees. The RF classifier utilizes a collection of individual trees built independently to determine the final output by the majority vote [63]. In contrast, the GBT classifier builds trees additively to reduce the bias of the previous tree, and then combines the output of all trees scaled by the learning rate to calculate the final output [64]. Parameters of both classifiers were manually tuned to optimize their classification performance. The following parameters were used in RF: the number of trees in the forest of 300, the minimum number of samples per leaf node of 85, the number of features to consider for the best split equal to the square root of total number of features, and class weights set to “balanced” in order to deal with the imbalanced dataset. The following parameters were used in GBT: the number of boosting stages of 650, the minimum number of samples per leaf node of 120, the number of features to consider for the best split equal to the square root of total number of features, the learning rate of 0.28, and the maximum depth of the individual regression estimators of 5.

## Figures and Tables

**Table 1:** Performance of machine learning in the prediction of drug synergistic effects. Two protocols are employed utilizing the random-split of the data and the tissue-based cross-validation. The performance of the Random Forest (RF) and Gradient Boosting Trees (GBT) classifiers is evaluated against the original AZ-DREAM Challenges data and the augmented dataset.

Classifier	Validation protocol	Dataset	ACC	TPR	FPR	PPV	AUC	MCC	F1-score
RF	Random-split	Original	0.754	0.787	0.358	0.879	0.802	0.392	0.831
		Augmented	0.757	0.788	0.347	0.882	0.809	0.402	0.832
	Tissue-based	Original	0.667	0.811	0.644	0.749	0.647	0.173	0.769
		Augmented	0.705	0.866	0.659	0.758	0.685	0.226	0.801
GBT	Random-split	Original	0.833	0.921	0.457	0.869	0.859	0.503	0.894
		Augmented	0.840	0.927	0.445	0.873	0.863	0.524	0.899
	Tissue-based	Original	0.716	0.930	0.803	0.734	0.688	0.176	0.815
		Augmented	0.736	0.940	0.743	0.750	0.734	0.260	0.828

ACC - accuracy, TPR - recall, FPR - false positive rate, PPV - precision, AUC - area under the receiver operating characteristic plot, MCC - Matthews correlation coefficient.

**Table 2:** Area under the receiver operating characteristic plot (AUC) scores for each fold in the tissue-based cross-validation. The performance of Random Forest (RF) and Gradient Boosting Trees (GBT) classifiers is reported for the original and the augmented AZ-DREAM Challenges datasets.

Classifier	Dataset	Breast tissue	Digestive system	Excretory system	Respiratory system	Other
RF	Original	0.574	0.628	0.650	0.636	0.746
	Augmented	0.613	0.640	0.664	0.658	0.849
GBT	Original	0.637	0.675	0.631	0.704	0.794
	Augmented	0.648	0.715	0.649	0.752	0.904

## Data Availability

All data are freely available at https://github.com/MengLiu90/Synergy-Data-Augmentation.
